# Mask-Induced Dermatitis Progressing to Chronic Seborrheic Inflammation: A Case Report

**DOI:** 10.7759/cureus.97095

**Published:** 2025-11-17

**Authors:** Mohammed Bin Rakan, Maha Rakan, Ibrahim Bin Rakan

**Affiliations:** 1 Department of Clinical Pharmacy, College of Pharmacy, King Saud University, Riyadh, SAU; 2 Pharmacy, SMC Hospital, Riyadh, SAU; 3 Emergency Medical Services, Prince Sultan bin Abdulaziz College for Emergency Medical Services (PSCEMS), Riyadh, SAU

**Keywords:** contact dermatitis, corticosteroid rebound, covid-19 pandemic, ketoconazole shampoo, mask-induced eczema, pharmacist case report, postauricular dermatitis, scalp eczema, tacrolimus

## Abstract

Prolonged mask use during the COVID-19 pandemic has been associated with an increase in dermatologic conditions, particularly irritant and allergic contact dermatitis. Mechanical friction, occlusion, and humidity contribute to skin barrier disruption and inflammation. Although most cases are self-limiting, a subset may evolve into chronic or steroid-dependent dermatoses. We report a case of a 23-year-old male healthcare professional who developed erythematous plaques behind the ear following continuous mask use. The lesions initially responded to topical betamethasone 0.05% cream but relapsed after tapering, evolving into chronic seborrheic inflammation with pruritus and scaling extending into the scalp and external auditory canal. Application of tacrolimus 0.1% ointment and fusidic acid cream aggravated the condition, reflecting persistent inflammation and barrier dysfunction. Clinical findings indicated steroid dependence and chronic seborrheic-type inflammation. Barrier-restoring measures and cautious corticosteroid withdrawal led to gradual improvement. This case highlights the multifactorial pathogenesis of chronic mask-related dermatitis and underscores the importance of barrier repair, prudent corticosteroid use, and interdisciplinary collaboration in managing persistent cases.

## Introduction

Dermatitis encompasses a broad spectrum of inflammatory skin disorders characterized by erythema, pruritus, and barrier impairment. Among these, contact dermatitis arises from exposure to irritants or allergens that trigger localized inflammation, often intensified by mechanical friction, sweat, occlusion, and prolonged humidity. During the COVID-19 pandemic, extensive and mandatory mask use led to a notable surge in mask-induced dermatitis (MID) among healthcare workers and the general population [1-3].

Recent epidemiological studies have shown that up to 40-60% of healthcare professionals reported new-onset or aggravated skin eruptions associated with mask use, particularly over areas of high friction and occlusion such as the nasal bridge, cheeks, and postauricular regions [4-6]. MID may manifest as irritant or allergic contact dermatitis, acneiform eruptions, pressure-related eczema, or mixed patterns resulting from continuous mechanical stress, sweat retention, and microclimate changes beneath the mask [7].

Prolonged barrier disruption has also been linked to the development of chronic or steroid-dependent dermatoses, including seborrheic and eczematous phenotypes [6,8]. This chronicity may be driven by altered skin microbiota, lipid imbalance, and recurrent corticosteroid exposure. Post-pandemic dermatologic surveillance has revealed that some cases evolve beyond acute irritation into persistent inflammation involving the scalp, retroauricular folds, and external auditory canal - regions highly susceptible to occlusion and humidity [6,9].

The present case describes the progression of mask-induced dermatitis into chronic seborrheic inflammation affecting the postauricular, scalp, and intra-auricular regions in a young healthcare professional. This report highlights the multifactorial etiology of chronic mask-related dermatoses and the importance of addressing barrier dysfunction and microbial dysbiosis in their management.

## Case presentation

**Figure 1:**
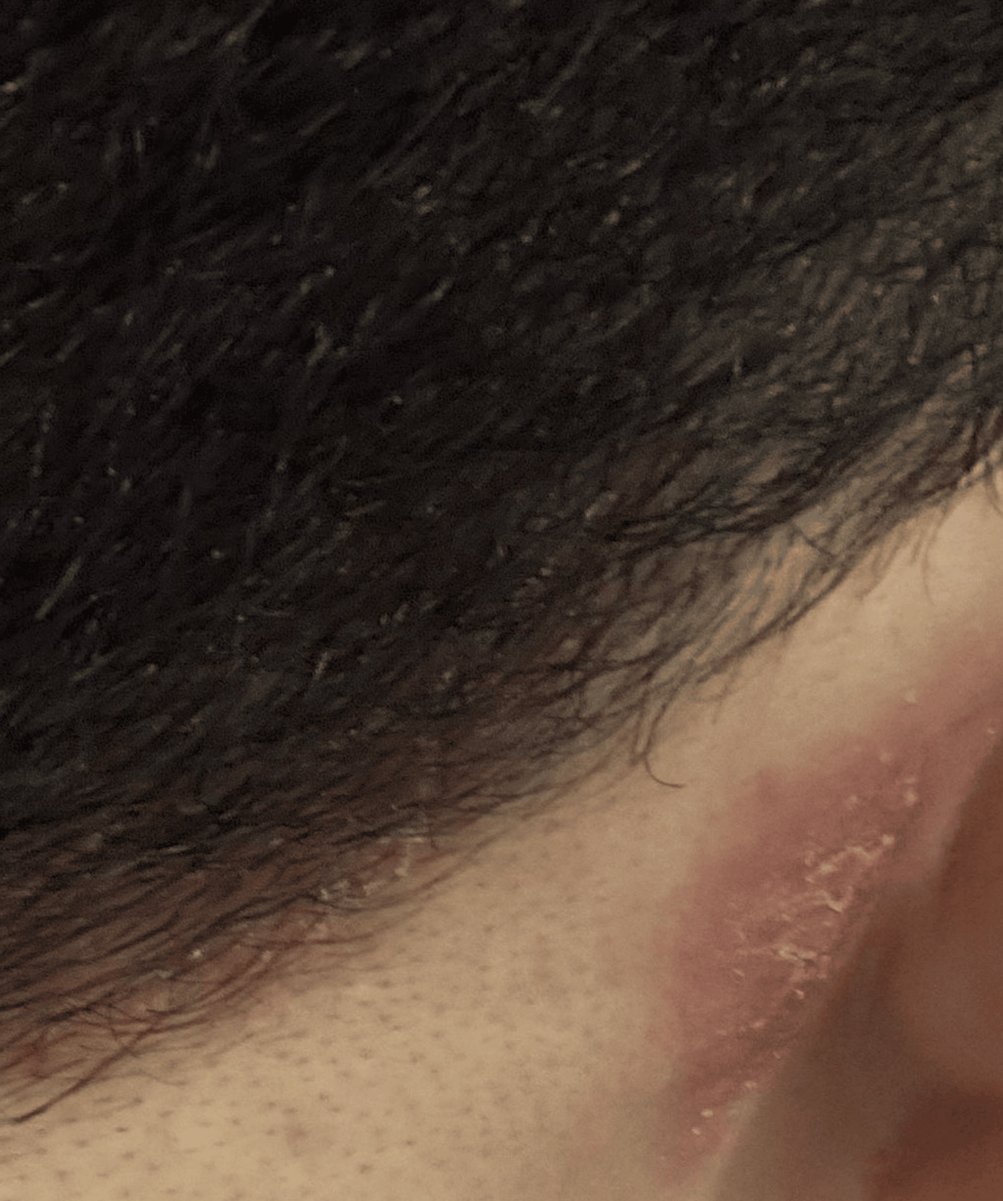
Initial presentation showing a well-demarcated erythematous and scaly plaque in the right postauricular region following prolonged mask use.

A 23-year-old male healthcare professional developed erythematous and pruritic plaques in the right postauricular region following prolonged mask use during the COVID-19 pandemic. The lesions appeared approximately three weeks after continuous mask use. On physical examination, erythematous and scaly plaques were observed extending from the retroauricular fold into the adjacent scalp and external auditory canal (Figure 1). The findings were consistent with an early irritant contact dermatitis related to prolonged occlusion and friction.

**Figure 2:**
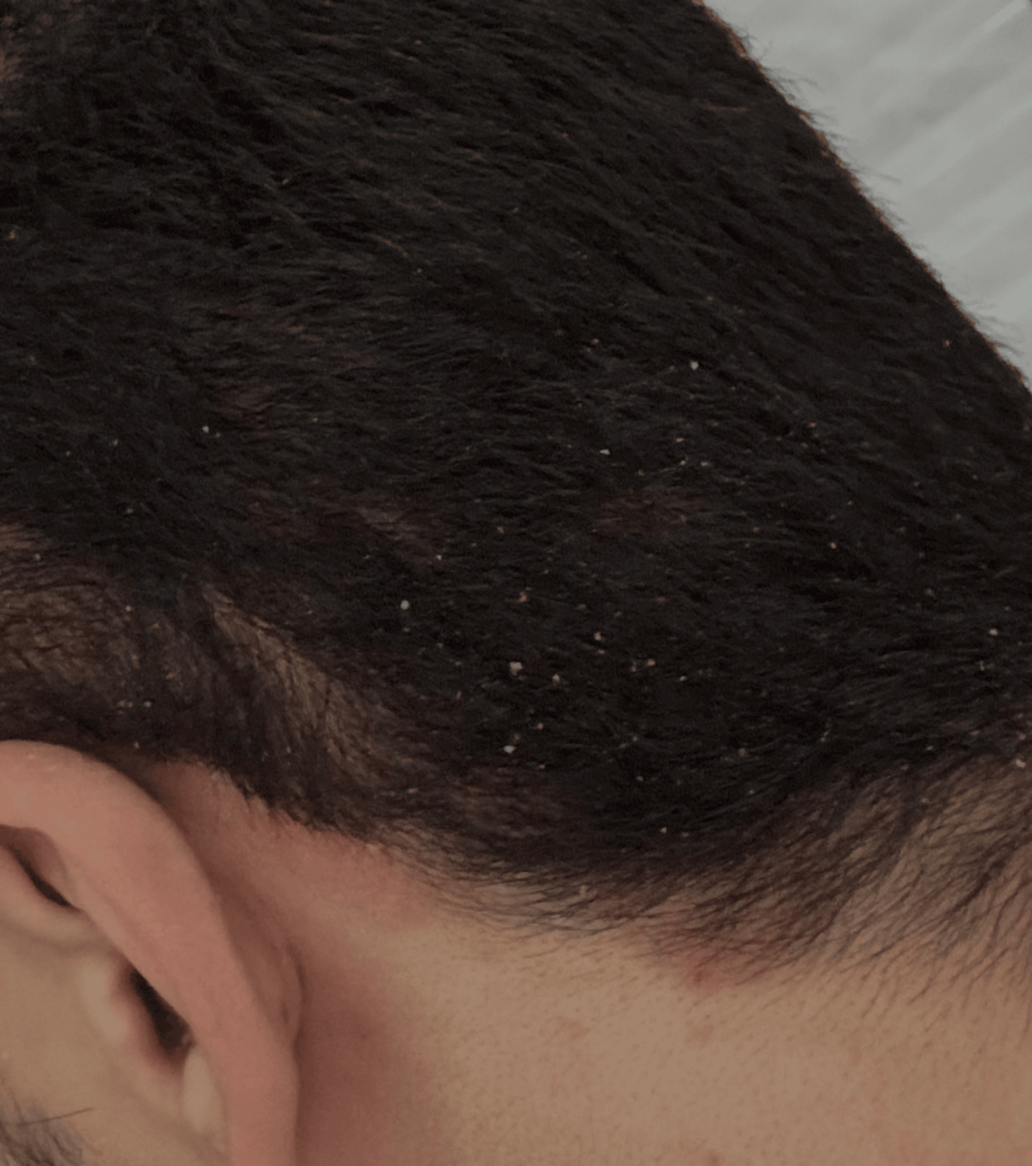
Clinical progression showing extension of the lesion into the adjacent scalp with greasy scaling and crust formation, findings consistent with seborrheic-type inflammation.

**Figure 3:**
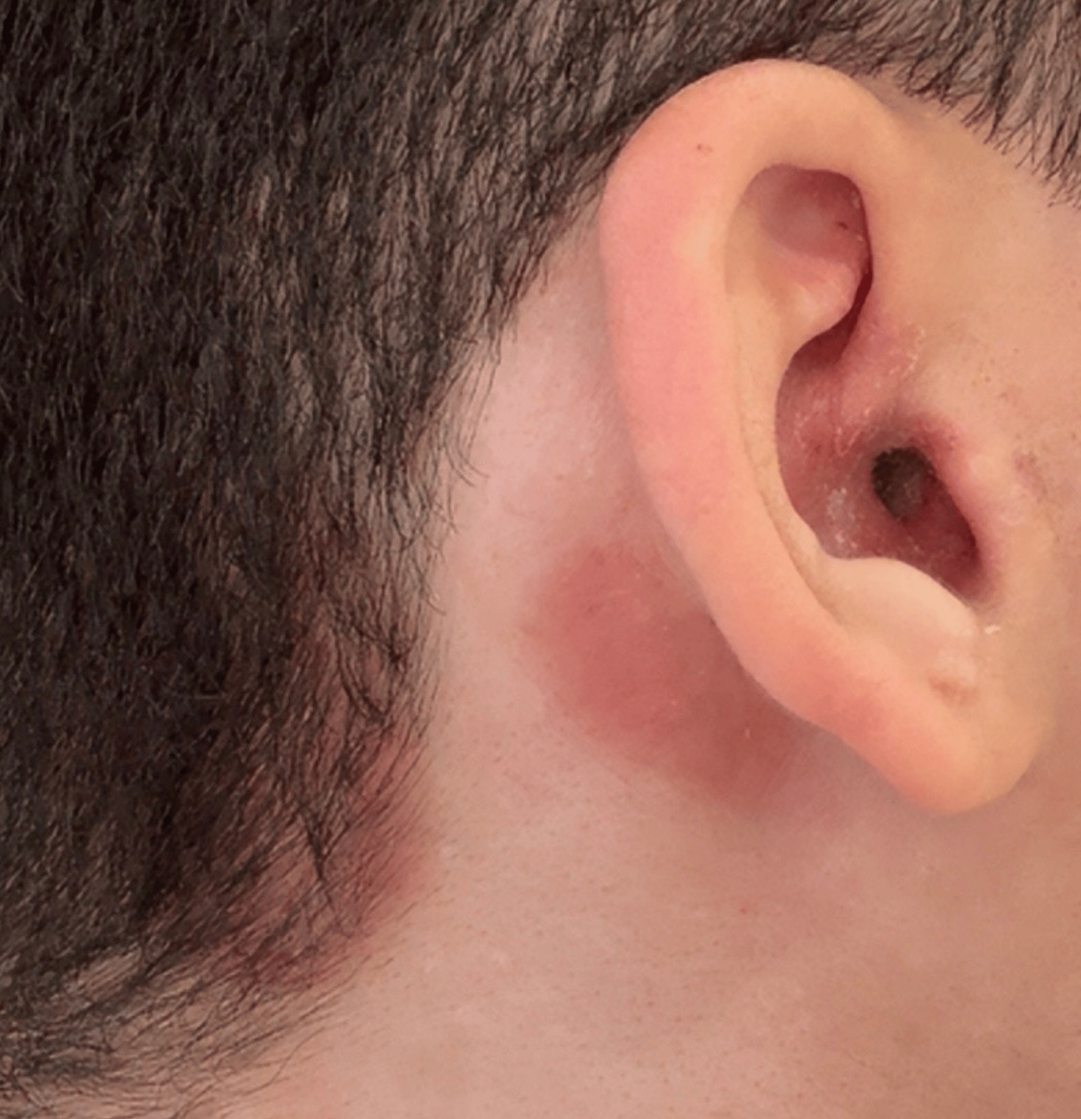
Partial response after treatment, showing residual erythema and scaling following intermittent topical corticosteroid therapy and barrier-repair measures.

Initial management with topical betamethasone 0.05% cream provided temporary improvement, with noticeable reduction of erythema and pruritus within five days. However, tapering the corticosteroid to once every three days led to partial recurrence after two weeks, characterized by persistent erythema and scaling (Figure 2). Subsequent application of tacrolimus 0.1% ointment and fusidic acid cream aggravated the condition, resulting in worsening erythema, intensified scaling and pruritus. Over the following weeks - approximately 10 weeks from onset - the clinical presentation evolved into a chronic seborrheic-type dermatitis with greasy scaling and crusting over the scalp (Figure 3).

The patient had no history of atopic dermatitis, psoriasis, or prior seborrheic dermatitis. No new hair or skin products were used during this period. Differential diagnoses, including psoriasis, allergic contact dermatitis, and superficial fungal infection, were considered and excluded based on lesion morphology, distribution pattern, lack of systemic symptoms, and the clear temporal association with mask use. Laboratory evaluation was not indicated, as the diagnosis was made clinically.

Management focused on discontinuing irritant products, maintaining gentle cleansing routines, and applying fragrance-free emollients twice daily. Short courses of low-potency topical corticosteroids were used intermittently. Patient education emphasized mask hygiene, alternating mask types, and minimizing strap tension. Over the following weeks, gradual improvement was observed, with reduction in erythema, scaling, and pruritus.

## Discussion

Prolonged mask use has been strongly associated with an increased incidence of dermatologic disorders, mainly irritant and allergic contact dermatitis, among healthcare professionals and the general population during the COVID-19 pandemic [1-4]. Mechanical friction, occlusion, retained moisture, and increased temperature beneath the mask are known to contribute to barrier disruption and inflammatory responses [5]. Although most cases are self-limited, a subset may evolve into chronic or steroid-dependent dermatoses due to repetitive irritation or inappropriate corticosteroid use [6,7].

Recent studies have identified MID as a multifactorial condition with overlapping features of seborrheic, eczematous, and acneiform eruptions [8]. Altered skin microbiota, particularly increased Malassezia colonization, combined with lipid imbalance and chronic occlusion, may contribute to a seborrheic-like phenotype in some patients [6,9]. In this case, the continuous mechanical friction behind the ear, coupled with prolonged topical corticosteroid exposure and abrupt tapering, likely precipitated barrier compromise and secondary seborrheic inflammation extending to the scalp and external auditory canal.

Management of chronic MID requires restoration of the epidermal barrier and cautious withdrawal of topical corticosteroids (TCS). Non-steroidal anti-inflammatory agents such as topical calcineurin inhibitors can serve as steroid-sparing alternatives; however, their use should be reserved for stabilized lesions to avoid irritation in compromised skin. Recent literature emphasizes the role of emollient-based barrier repair, patient education on mask hygiene, and alternating mask types to reduce friction and occlusion-related damage [6-9].

This case underscores the evolving spectrum of post-pandemic dermatologic complications and the need for multidisciplinary collaboration between dermatologists, pharmacists, and occupational health teams to optimize treatment outcomes and prevent chronic steroid-dependent dermatoses.

## Conclusions

This case highlights the potential for MID to transition into chronic seborrheic-type inflammation, particularly when prolonged topical corticosteroid exposure and abrupt tapering compromise the skin barrier. The clinical evolution observed supports the multifactorial pathogenesis of chronic mask-related dermatoses, in which barrier dysfunction, microbial dysbiosis, and lipid imbalance play pivotal roles.

Comprehensive management should prioritize early recognition, patient education, and restoration of epidermal barrier integrity to prevent recurrent or steroid-dependent inflammation. Gradual tapering of topical corticosteroids and the judicious use of non-steroidal anti-inflammatory alternatives are essential to minimize rebound phenomena.

This case underscores the importance of cautious corticosteroid stewardship and contributes to the growing body of literature addressing the long-term dermatologic consequences of prolonged mask use in the post-pandemic era.
